# Catechol-O-methyltransferase (COMT) Genotype Affects Age-Related Changes in Plasticity in Working Memory: A Pilot Study

**DOI:** 10.1155/2014/414351

**Published:** 2014-03-19

**Authors:** Stephan Heinzel, Thomas G. Riemer, Stefanie Schulte, Johanna Onken, Andreas Heinz, Michael A. Rapp

**Affiliations:** ^1^Department of Psychology, Humboldt-Universität zu Berlin, Rudower Chaussee 18, 12489 Berlin, Germany; ^2^Department of Psychiatry and Psychotherapy, Campus Charité Mitte, Charité–Universitätsmedizin Berlin, Charitéplatz 1, 10117 Berlin, Germany; ^3^Social and Preventive Medicine, University of Potsdam, Am Neuen Palais 10, 14469 Potsdam, Germany; ^4^Cluster of Excellence NeuroCure, Charité-Universitätsmedizin Berlin, Charitéplatz 1, 10117 Berlin, Germany

## Abstract

*Objectives*. Recent work suggests that a genetic variation associated with increased dopamine metabolism in the prefrontal cortex (catechol-O-methyltransferase Val158Met; COMT) amplifies age-related changes in working memory performance. Research on younger adults indicates that the influence of dopamine-related genetic polymorphisms on working memory performance increases when testing the cognitive limits through training. To date, this has not been studied in older adults.* Method*. Here we investigate the effect of COMT genotype on plasticity in working memory in a sample of 14 younger (aged 24–30 years) and 25 older (aged 60–75 years) healthy adults. Participants underwent adaptive training in the *n*-back working memory task over 12 sessions under increasing difficulty conditions. * Results*. Both younger and older adults exhibited sizeable behavioral plasticity through training (*P* < .001), which was larger in younger as compared to older adults (*P* < .001). Age-related differences were qualified by an interaction with COMT genotype (*P* < .001), and this interaction was due to decreased behavioral plasticity in older adults carrying the Val/Val genotype, while there was no effect of genotype in younger adults.* Discussion*. Our findings indicate that age-related changes in plasticity in working memory are critically affected by genetic variation in prefrontal dopamine metabolism.

## 1. Introduction

Working memory performance declines with normal aging [1], and evidence from animal models [2], human imaging [3], and pharmacological challenge studies [4] suggests that age-related declines in working memory are associated with decreased dopaminergic neurotransmission. The enzyme catechol-O-methyltransferase (COMT; EC 2.1.1.6) degrades the neurotransmitters dopamine, epinephrine, and norepinephrine, and a functional polymorphism in the COMT gene (Val158Met) accounts for a fourfold variation in enzyme activity, resulting in a substantial decrease in dopamine availability in carriers of the Val/Val genotype [5]. The prefrontal cortex, a region which is frequently associated with executive function and working memory, seems to be particularly prone to an increased COMT activity. It has been argued that this is due to the lack of the dopamine transporter (DAT) and the resulting particular importance of COMT for the degradation of dopamine [6]. There is initial experimental evidence that the COMT polymorphism affects working memory performance as well as associated brain activation in healthy older adults [7, 8], and recent evidence suggests that COMT effects are amplified in older as compared to younger adults [9]. Overall, however, evidence is conflicting: a population-based study detected the effects of COMT on maintenance and updating in working memory using a letter-number sequencing task [10], while recent cross-sectional studies in younger [11, 12] and older adults [13] detected no effect of COMT genotype on working memory functions and general cognitive ability “*g*”, respectively.

It has been assumed that the influence of polymorphisms in genes related to the dopamine system on working memory performance may be more pronounced in a training context than in single-assessment performance scores, possibly because the influence of confounding factors is reduced [14]. Indeed, two recent working memory training studies conducted with younger adults (range 20–31 years) revealed post- but not pretraining differences between carriers of different DAT [15] and LIM homeobox transcription factor 1, alpha (LMX1A) [16] genotypes.

The testing-the-limits approach [17, 18] has been introduced in cognitive aging research as a method to use adaptive training procedures in order to estimate behavioral (cognitive) plasticity, as measured, for example, in individual differences in the maximum gain that can be induced during behavioral training in a cognitive task. However, effects of dopaminergic neurotransmission on age-related changes in behavioral plasticity in working memory, that is, training-related increases using adaptive training studies, have not hitherto been studied. Here we investigate the effect of COMT genotype on plasticity in working memory in younger and older healthy adults, using an adaptive (testing-the-limits) training procedure in the *n*-back working memory task over 12 sessions. We hypothesized that (i) behavioral plasticity in younger adults would be larger than in older adults, (ii) carriers of the Val/Val genotype of the COMT gene would show decreased behavioral plasticity in working memory, as compared to carriers of a Met allele, and (iii) the effect of COMT genotype on plasticity would be larger in older than in younger adults. Given reports that effects of COMT genotype on working memory performance may be moderated by gender [19] and educational attainment [20], we controlled for these variables in our study.

## 2. Methods

### 2.1. Subjects

15 younger (range 24–30 years) and 33 older (range 60–75 years) healthy adults were recruited from the community. One participant in the younger group and 6 participants in the older group denied blood sampling, and 2 older participants dropped out of the training program. Therefore, the final analysis sample consisted of 14 younger and 25 older participants (see Table 1). All older participants performed within the age-related normal range (±1 SD) for tests of processing speed, executive functions, memory, and attention from the CERAD (Consortium to Establish a Registry for Alzheimer's Disease [21]) neuropsychological battery. Participants were task naïve (i.e., they never performed the task prior to our study) and had no history of psychiatric or neurological diseases. The study was approved by the local Ethical Committee and conducted in accordance with Declaration of Helsinki principles (1964). Written informed consent was obtained from all participants.

### 2.2. Working Memory Assessment

All participants completed 12 sessions (45 minutes each) of adaptive *n*-back working memory training over a period of 4 consecutive weeks (three sessions per week). Before the first training session, 20 mL of blood was collected and neuropsychological testing was conducted. The training task consisted of a computerized numerical version of the *n*-back paradigm [22], during which white digits ranging from 0 to 9 were visually presented for the duration of 500 ms in the center of an otherwise black screen in a random sequence. Each block consisted of 20 to 28 trials including 5 to 7 targets. Task difficulty was adaptively increased by reducing the interstimulus interval and by increasing the memory load from 2-back up to 5-back (for specific details of the training procedure please see [23]). Participants began training at session 1 with difficulty level 1 (0-, 1-, and 2-back; interstimulus interval = 1800 ms). If a subject successfully completed the first run (that is 3 blocks of 0-back, 3 blocks of 1-back, and 3 blocks of 2-back) with a hit rate of 80% or above within each block and with no false alarms, the next difficulty level was introduced in the following run. From level 1 to level 5, interstimulus interval gradually decreased from 1800 to 1000 ms in steps of 200 ms. At level 6, the next *n*-level was introduced (3-back) and 1-back was removed; that is, participants completed 3 blocks of each 0-, 2-, and 3-back. In addition, interstimulus interval was set back to 1800 ms. This procedure continued until 5-back was introduced at level 16.

### 2.3. Neuropsychological Assessment

For neuropsychological screening, neuropsychological tests were selected for measuring short-term memory (Digit Span Forward, Digit Span Backward), processing speed (Digit Symbol), episodic memory (CERAD Delayed Recall), executive functions (Verbal Fluency), and reasoning (Raven's SPM, Figural Relations). 

*Short-Term Memory Tasks.* To obtain an estimate of each participant's short-term memory capacity, Digit Span Forward and Backward from the Wechsler Adult Intelligence Scale (WAIS, [24]) were administered. Two trials of each list length were presented. If participants failed to repeat both trials of a certain list length, the assessment of this task was terminated. The score used in the following analyses was determined by the length of the longest correctly repeated trial.

*Processing Speed Task.* The Digit Symbol Substitution subtest (Digit Symbol) of the WAIS [24] was included to assess mental processing speed and attention. In Digit Symbol, participants were asked to copy symbols as quickly as possible into empty boxes located below a random sequence of numbers ranging from 1 to 9 according to a specific coding key. The score used for analyses was the number of correct symbols completed within 60 seconds.

*Episodic Memory Task.* As a measure of episodic memory, all participants performed the memory task from the neuropsychological test battery of the Consortium to Establish a Registry for Alzheimer's Disease (CERAD; [21]). Participants were asked to remember 10 words that were presented to them sequentially three times in varying order and recall the words after a delay. For further analyses, the number of correctly recalled items after a delay of 15 minutes was used (CERAD Delayed Recall).

*Executive Functions Task.* Verbal Fluency requires the ability to generate words while monitoring previously recalled words and following specific rules. Verbal Fluency was assessed by a German version of the Controlled Oral Word Association Test (COWAT; [25]). Participants were asked to generate as many words as possible starting with the letter “S” within 60 seconds (not including proper names or names of places and cities).

*Abstract Reasoning Tasks*. Abstract reasoning abilities were measured by Raven's Standard Progressive Matrices (Raven's SPM; [26]) and by the Figural Relations subtest of a German intelligence test (Leistungspruef system; [27]). To solve these tasks, participants were required to identify patterns of nonverbal symbols. In Raven's SPM, they were instructed to find a matching item to complete a pattern, while in the Figural Relations they had to mark the nonmatching item of a pattern of symbols. Both reasoning tasks were timed and the scores were derived from the number of correct items accomplished within 7.5 minutes (Raven's SPM) or 3 minutes (Figural Relations), respectively.

### 2.4. Blood Sampling and Analyses

Blood was sampled and stored at a temperature below −20°C. Genotype analyses were conducted using commercially available kits (Biologis, Frankfurt, Germany). Specifically, whole blood was thawed, DNA was extracted, and exon 4 of the COMT gene was amplified using reverse PCR and genotyped using pyrosequencing. Overall, there were 5 carriers of Met/Met, 20 heterozygotes, and 14 carriers of the Val/Val genotype. The distribution was not significantly different from Hardy-Weinberg equilibrium (*χ*² = .27, *P* = .603). Carriers of either Val/Met or Met/Met COMT genotype were classified into one group (any Met) and contrasted with Val/Val carriers (see [9, 10] for a similar approach).

### 2.5. Statistics

Statistical analyses were performed with SPSS version 17.0 (SPSS Inc., Chicago, IL). For our hypotheses, we performed a 2 (older versus younger) × 2 (Val/Val versus any Met) × 12 (training session 1 through 12) mixed model between-group within-subjects analysis of variance with *α* set to .05 and *β* set to .8. An a priori power analysis with a minimum cell size of *N* = 3, a minimum detectable difference of two *n*-back levels for main effects (hypotheses  1 and 2), and one level for interaction effects (hypothesis  3) revealed a power of >.80 to detect main effects and of >.60 to detect within-subjects interactions (PASS software, NCSS Inc., Kaysville, Utah).

## 3. Results

### 3.1. Differences between Genotype Groups at Pretest

Demographic characteristics and neuropsychological test performance of the study sample, as a function of age and genotype, are reported in Table 1. Within the younger group, there were no significant differences between carriers of the Val/Val COMT genotype and Met allele carriers with respect to age, gender distribution, years of education, initial *n*-back performance, mental status, and performance in neuropsychological tests (all *P*s > .16).

Within the older group, no significant differences between Val/Val and any Met COMT genotypes were found with respect to age, gender distribution, years of education, mental status, and initial *n*-back performance (all *P*s > .09). Met allele carriers showed a trend towards better performance in Raven's SPM compared to Val/Val carriers (*T*_23_ = 1.94, *P* = .065). In all other neuropsychological tests, no significant differences were found between the genotype groups (all *P*s > .18).

### 3.2. Training Gains, Age, and COMT Genotype

After 12 sessions of *n*-back training, younger carriers of the Val/Val genotype achieved *n*-back difficulty level 14 on average (*M* = 14.00, SD = 4.36), Met allele carriers reached level 12 (*M* = 11.55, SD = 2.94). *T*-tests showed that this difference was not significant (*T*_12_ = 1.17, *P* = .265). Older Val/Val carriers accomplished level 4 (*M* = 4.45, SD = 1.57) and Met allele carriers reached level 6 (*M* = 6.07, SD = 2.06, *T*_23_ = 2.16, *P* = .042).

**Figure 1:**
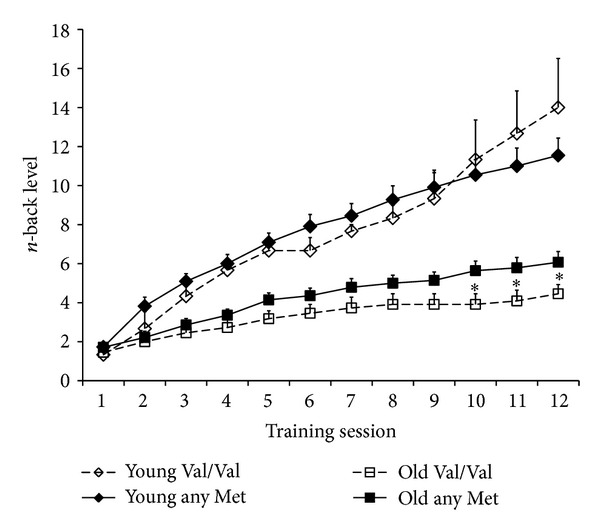
Behavioral plasticity (level attained in each session) in younger (diamonds) and older adults (squares) as a function of genotype (dotted line represents Val/Val; continuous line represents any Met). Error bars reflect standard errors of the mean. Significant differences in *n*-back levels within age groups are marked by asterisks.

To investigate the influence of age and COMT genotype on the level progression through adaptive training, a 2 (older versus younger) × 2 (Val/Val versus any Met) × 12 (training session one through 12) mixed model between-group within-subjects analysis of variance was conducted and revealed a main effect of age group (*F*_1,35_ = 44.71, *P* < .001) but not of genotype (*F*_1,35_ = .95, *P* = .337). There was a large within-subjects effect for training session (*F*_11,385_ = 151.31, *P* < .001), which was qualified by both a training × age group (*F*_11,385_ = 37.42, *P* < .001) and a training × age group × genotype interaction (*F*_11,385_ = 4.53, *P* < .001). As shown in Figure 1, these interactions were due to an increased overall plasticity in younger as compared to older adults and a decreased plasticity in older carriers of the Val/Val genotype as compared to older carriers of any Met allele.

To control for effects of gender and education, a mixed model between-group within-subjects analysis of covariance was conducted. Again, a main effect of age group was revealed (*F*_1,33_ = 35.79, *P* < .001), indicating that younger adults achieve higher training gains in *n*-back compared to older adults irrespective of gender and education. No main effects of genotype (*F*_1,33_ = .88, *P* = .357), gender (*F*_1,33_ = 1.03, *P* = .318), and education (*F*_1,33_ = .47, *P* = .500) were found. Again, significant training × age group (*F*_11,363_ = 29.73, *P* < .001) and training × age group × genotype interactions were revealed (*F*_11,363_ = 2.96, *P* = .001).

A separate 2 (genotype) × 12 (training session) analysis of variance within the older group revealed a significant training × genotype effect (*F*_11,253_ = 2.57, *P* = .004), suggesting that the slope of level progression is less steep in older Val/Val carriers compared to older Met allele carriers.

In older adults, post hoc *t*-tests showed that genotype-related differences in the level progression were only significant in the last quarter of the training program (session 10: *T*_23_ = 2.37, *P* = .027; session 11:* T*_23_ = 2.19, *P* = .039; session 12: *T*_23_= 2.16, *P* = .042, see Figure 1). In younger participants, no significant genotype-related differences in the level progression were revealed by post hoc *t*-tests (all *Ps* > .26).

## 4. Discussion

Our results indicate that differences in dopamine metabolism, as related to a polymorphism (Val158Met) of the COMT gene, may affect working memory plasticity in older adults. Both younger and older adults exhibited sizeable behavioral plasticity, which was larger in younger as compared to older adults. However, these age-related differences were qualified by an interaction with COMT genotype. This interaction was due to decreased behavioral plasticity in older adults homozygous for the Val allele, as compared to older Met allele carriers. Specifically, our data suggest that older Val/Val carriers can increase their *n*-back performance at a lower rate (less steep slope in level progression; see Figure 1) and reach their maximum performance at a lower difficulty level compared to Met allele carriers. We did not detect a similar effect in younger adults. Our findings are consistent with previous results [9], which showed that effects of the COMT genotype are larger in older than in younger adults. These age-related differences have been interpreted within the framework of a nonlinear (inverse U-shaped) relationship between prefrontal dopamine availability and executive cognitive performance. Due to an age-related decrease of dopaminergic neurotransmission, older adults represent the left and relatively steep section of this proposed dopamine/performance inverse U-curve. Therefore, an additional reduction in dopamine availability caused by an increased enzymatic activity of the Val/Val COMT genotype seems to have a stronger impact on working memory performance in older compared to younger adults. The testing-the-limits approach [17, 18] employed in our study suggests that the behavioral malleability of working memory functions in older adults critically depends on dopamine metabolism.

No differences in neuropsychological test performance were detected at baseline assessment. However, the sensitivity of the testing-the-limits approach [17] may have enabled us to detect differences even in a small sample since adaptive training has been suggested to magnify individual differences in cognitive performance [28]. Therefore, divergent nonfindings from other studies may be due to the one-time assessment of cognitive function and also due to the wide age range typically present in these samples [10, 11, 13].

Even though significant effects of age, training, and genotype were detected in the current study, sample size is a limitation and further independent replication is needed. Another point to consider is that the effects of the COMT genotype have been shown to interact with certain variants of other genes associated with dopaminergic neurotransmission, such as the DAT gene [15, 29, 30] and the D-amino acid oxidase activator (DAOA [G72]) gene [31]. Furthermore, interactions between genotypes and phenomena of age-related cognitive decline (see [32] for review), such as loss of gray and white matter volume [33] and changes in brain functioning and connectivity [34], could be investigated in regard to working memory plasticity in future research. Another valuable extension of the current study would be to include additional samples of different age groups within the age range of below 20, between 30 and 60, and above 75 years to gain a more profound understanding of dopamine-related genetic influences on plasticity across the entire lifespan. Finally, as suggested by Slagter [35], future working memory training studies would benefit from longer training periods (e.g., 25 training sessions) and follow-up measurements to further understand the different shapes of learning curves across different age and genotype groups and to test the stability of training effects over time.

## 5. Conclusion

Working memory declines with age [1], and age-related decline of, for example, cortical D1 receptors [3] has been shown to correlate with decline in cognitive function. Focusing on a genetic variation in dopamine metabolism, we detected a potential link between differences in dopaminergic neurotransmission and behavioral plasticity in working memory in the elderly. Further understanding of the influences of genetic variations on working memory plasticity could have strong implications on designing individually tailored training programs in healthy and cognitively impaired older adults.

## Figures and Tables

**Table 1:** Characteristics of the sample at baseline assessment. Unless otherwise indicated, values represent means ± standard deviations.

	Younger (*N* = 14)			Older (*N* = 25)		
	Val/Val (*N* = 3)	Any Met (*N* = 11)	*T*_(12)_ (*P*)	Val/Val (*N* = 11)	Any met (*N* = 14)	*T*_(23)_ (*P*)
Age	26.00 ± 1.00	26.09 ± 2.17	0.069 (0.946)	67.36 ± 4.34	64.64 ± 3.37	1.77 (0.090)
Gender *N* (female/male)	1/2	8/3	*χ* ^2^(14.1) = 1.59, *P* = 0.224	5/6	7/7	*χ* ^2^(25.1) = 0.05, *P* = 0.821
Years of education	18.67 ± 0.58	18.18 ± 1.74	0.47 (0.651)	15.46 ± 3.15	16.88 ± 3.62	1.10 (0.281)
Minimental state examination total score	30.00 ± 0.00	29.91 ± 0.30	0.51 (0.621)	29.27 ± 1.01	29.64 ± 0.84	1.00 (0.328)
*n*-back performance (% correct)	96.23 ± 2.39	97.73 ± 1.81	1.17 (0.266)	88.47 ± 5.96	91.53 ± 5.29	1.36 (0.188)
Digit Span Forward	7.67 ± 0.58	6.91 ± 0.83	1.46 (0.169)	6.64 ± 0.81	7.14 ± 1.03	1.34 (0.194)
Digit Span Backward	6.67 ± 0.58	6.00 ± 0.89	1.20 (0.252)	5.27 ± 1.42	5.21 ± 1.25	0.11 (0.914)
Digit Symbol	46.00 ± 5.29	46.00 ± 7.59	0.00 (1.00)	32.73 ± 6.80	32.79 ± 6.66	0.02 (0.983)
CERAD Delayed Recall	9.00 ± 1.73	9.63 ± 0.50	1.16 (0.269)	7.91 ± 1.87	8.07 ± 1.77	0.22 (0.826)
Verbal Fluency	18.33 ± 1.53	20.55 ± 4.25	0.86 (0.404)	18.00 ± 6.99	18.14 ± 5.48	0.06 (0.955)
Figural Relations	26.67 ± 2.52	24.45 ± 2.70	1.27 (0.227)	18.36 ± 3.93	20.00 ± 3.82	1.05 (0.305)
Raven's SPM	22.00 ± 2.65	22.36 ± 1.24	0.14 (0.889)	14.91 ± 4.18	17.79 ± 3.26	1.94 (0.065)
